# Supplementary material for: Multiplexed imaging of immune cells in staged multiple sclerosis lesions by mass cytometry
Source: eLife. 2019 Aug 1;8:e48051. doi: 10.7554/eLife.48051 (PMC6707785; doi:10.7554/eLife.48051)
Supplement: Transparent reporting form [file elife-48051-transrepform.pdf]

eLIFE
Westbrook Centre, Milton Road
Cambridge CB4 1YG
UK
P 01223 855340
W elifesciences.org
T @elife

eLife's transparent reporting form

We encourage authors to provide detailed information *within their submission* to facilitate the interpretation and replication of experiments. Authors can upload supporting documentation to indicate the use of appropriate reporting guidelines for health-related research (see EQUATOR Network), life science research (see the BioSharing Information Resource), or the ARRIVE guidelines for reporting work involving animal research. Where applicable, authors should refer to any relevant reporting standards documents in this form.

If you have any questions, please consult our Journal Policies and/or contact us: editorial@elifesciences.org.

Sample-size estimation

- You should state whether an appropriate sample size was computed when the study was being designed
- You should state the statistical method of sample size computation and any required assumptions
- If no explicit power analysis was used, you should describe how you decided what sample (replicate) size (number) to use

Please outline where this information can be found within the submission (e.g., sections or figure legends), or explain why this information doesn't apply to your submission:

We have applied a new technique to study the human brain. Therefore, there is no precedent in the literature. It follows that the variability of the data generated using this technique in human brain is unknown. For these reasons, no power analysis was performed.
This is a pilot study. The data generated in this work is from a single biological replicate (one MS case and one control case).

Sample size:

- The samples size of the brain fields analyzed for the correlation study is n=11 for nuclei, n=7 for $CD3^+$ cells, n=14 for PLP, n=15 for HLA and n=15 for CD68. *This is stated in the legend of figure 1 and Figure 1 – Figure Supplement 4.*
- All lesions identified in two tissue blocks from one case of severe rebound multiple sclerosis activity after withdrawal from a monoclonal antibody therapy were analyzed. Two active lesions from block CL3a and 3 more from block CR4a were ablated. Six mixed active-inactive lesions (3 from block CL3a and 3 from block CR4a) and 2 normal appearing white matter (1 from block CL3a and 1 from block CR4a) were ablated. *The number and types of lesions analyzed is reported in table 1.*

Replicates

- You should report how often each experiment was performed
- You should include a definition of biological versus technical replication
- The data obtained should be provided and sufficient information should be provided to indicate the number of independent biological and/or technical replicates
- If you encountered any outliers, you should describe how these were handled
- Criteria for exclusion/inclusion of data should be clearly stated
- High-throughput sequence data should be uploaded before submission, with a private link for reviewers provided (these are available from both GEO and ArrayExpress)

Please outline where this information can be found within the submission (e.g., sections or figure legends), or explain why this information doesn't apply to your submission:

Biological replicate.
This is a pilot study. The data generated in this work is from a single biological replicate (one MS case and one control case).

Statistical reporting

- Statistical analysis methods should be described and justified
- Raw data should be presented in figures whenever informative to do so (typically when N per group is less than 10)
- For each experiment, you should identify the statistical tests used, exact values of N, definitions of center, methods of multiple test correction, and dispersion and precision measures (e.g., mean, median, SD, SEM, confidence intervals; and, for the major substantive results, a measure of effect size (e.g., Pearson's r, Cohen's d)
- Report exact p-values wherever possible alongside the summary statistics and 95% confidence intervals. These should be reported for all key questions and not only when the p-value is less than 0.05.

Please outline where this information can be found within the submission (e.g., sections or figure legends), or explain why this information doesn't apply to your submission:

- Correlation analysis of the density of nuclei or the density of cells or the % stained area detected either by IF or by IMC was performed with the nonparametric Spearman rank correlation. Differences were considered significant at $p < 0.05$. *This is stated in the methods section of the submission.*
Correlation coefficient: for nuclei, r=0.9182, p=0.0002; for $CD3^+$ cells, r=0.8929, p=0.01; for PLP, r=0.9544, p<0.0001; for HLA, r=0.9794, p<0.0001; for CD68, r=0.9051, p<0.0001. *This is stated in the legend of figure 1 and Figure 1 – Figure Supplement 4.*
- The samples size of the brain fields analyzed for the correlation study is n=11 for nuclei, n=7 for $CD3^+$ cells, n=14 for PLP, n=15 for HLA and n=15 for CD68. *This is stated in the legend of figure 1 and Figure 1 – Figure Supplement 4.*
- No statistical analysis of the density and distance map was performed because although multiple brain lesions were analyzed, these are from a single biological replicate.

(For large datasets, or papers with a very large number of statistical tests, you may upload a single table file with tests, Ns, etc., with reference to sections in the manuscript.)

Group allocation

- Indicate how samples were allocated into experimental groups (in the case of clinical studies, please specify allocation to treatment method); if randomization was used, please also state if restricted randomization was applied
- Indicate if masking was used during group allocation, data collection and/or data analysis

Please outline where this information can be found within the submission (e.g., sections or figure legends), or explain why this information doesn't apply to your submission:

Allocation into experimental groups is not applicable because we analyzed a single biological replicate (one MS case and one control case).
Groups of lesion types were defined according to the definition outlined in the methods section of the submission. The groups included:
White matter of control
Normal-appearing white matter
Peri-plaque white matter
(p)reactive lesion
Active lesion
Mixed active-inactive lesion

Additional data files ("source data")

- We encourage you to upload relevant additional data files, such as numerical data that are represented as a graph in a figure, or as a summary table
- Where provided, these should be in the most useful format, and they can be uploaded as "Source data" files linked to a main figure or table
- Include model definition files including the full list of parameters used
- Include code used for data analysis (e.g., R, MatLab)
- Avoid stating that data files are "available upon request"

Please indicate the figures or tables for which source data files have been provided:

- Source data file is provided for figure 7: Figure 7 – Source Data 1.
- The Jupyter notebook code used to generate the density and distance maps (Figure 7 of the submission) and the R code to generate the PHATE plots (Figure 8 of the submission), are accessible on the STTARR GitHub public repository, https://github.com/STTARR
